# DC Electric Fields Promote Biodegradation of Waterborne Naphthalene in Biofilter Systems

**DOI:** 10.1021/acs.est.4c02924

**Published:** 2024-10-01

**Authors:** Jinyao He, Jose Carlos Castilla-Alcantara, Jose Julio Ortega-Calvo, Hauke Harms, Lukas Y. Wick

**Affiliations:** † Department of Applied Microbial Ecology, Helmholtz Centre for Environmental Research UFZ, Leipzig 04318, Germany; ‡ Instituto de Recursos Naturales y Agrobiología de Sevilla (IRNAS-CSIC), Avda. Reina Mercedes 10, Seville E-41012, Spain

**Keywords:** biodegradation, biofiltration, direct electric current, contaminant, electro-kinetics, electroosmosis, hydraulic flow, naphthalene

## Abstract

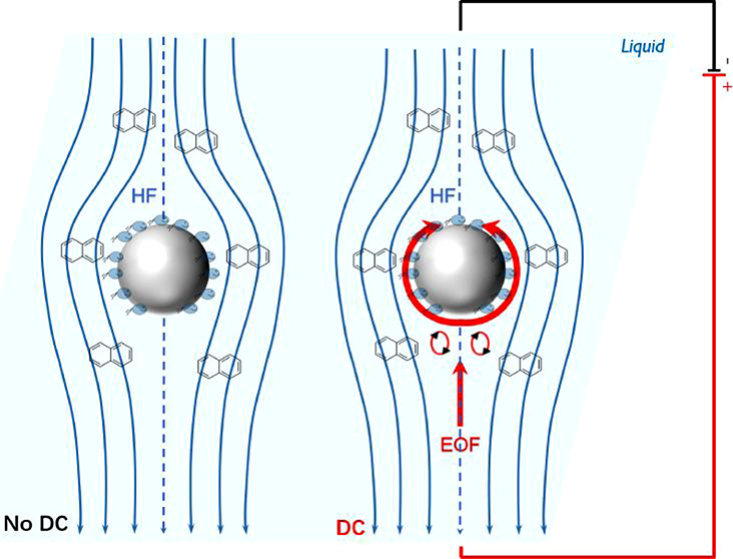

Biofiltration is a simple and low-cost method for the cleanup of contaminated water. However, the reduced availability of dissolved chemicals to surface-attached degrader bacteria may limit its efficient use at certain hydraulic loadings. When a direct current (DC) electric field is applied to an immersed packed bed, it invokes electrokinetic processes, such as electroosmotic water flow (EOF). EOF is a surface-charge-induced plug-flow-shaped movement of pore fluids. It acts at a nanometer distance above surfaces and allows the change of microscale pressure-driven flow profiles and, hence, the availability of dissolved contaminants to microbial degraders. In laboratory percolation columns, we assessed the effects of a weak DC electric field (*E* = 0.5 V·cm^–1^) on the biodegradation of waterborne naphthalene (NAH) by surface-attached *Pseudomonas fluorescens* LP6a. To vary NAH bioavailability, we used different NAH concentrations (*C*_0_ = 2.7, 5.1, or 7.8 × 10^–5^ mol·L^–1^) and Darcy velocities typical for biofiltration ( = 0.2–1.2 × 10^–4^ m·s^–1^). In DC-free controls, we observed higher specific degradation rates (*q*_c_) at higher NAH concentrations. The *q*_c_ depended on , suggesting bioavailability restrictions depending on the hydraulic residence times. DC fields consistently increased *q*_c_ and resulted in linearly increasing benefits up to 55% with rising hydraulic loadings relative to controls. We explain these biodegradation benefits by EOF-altered microscale flow profiles allowing for better NAH provision to bacteria attached to the collectors even though the EOF was calculated to be 100–800 times smaller than bulk water flow. Our data suggest that electrokinetic approaches may give rise to future technical applications that allow regulating biodegradation, for example, in response to fluctuating hydraulic loadings.

## Abstract 2

Superimposition of electric field-induced electrokinetic effects and hydraulic loadings promote the bioavailability and biodegradation of waterborne NAH in a biofiltration system.

## Introduction

Biofiltration is a simple and low-cost method for water cleanup that is often used for treating runoff and process waters or the purification of drinking water.^1,2^ It enables the physicochemical retention of particles and promotes biodegradation of waterborne contaminants by surface-attached microbes, respectively.^3^ Interweaving biotic and abiotic factors thereby drives biodegradation, including the abundance and activity of degraders, filter media, inflow characteristics, contaminant load, or hydraulic residence times (defined by the ratio of the pore volume of the bed and the flow rate). Efficient biodegradation requires sufficient contaminant bioavailability, that is, the interplay of contaminant mass transfer to cells and their degradation capacity.^4^ The ratio of the contaminant mass transfer rate and the biodegradation rate can be estimated by the bioavailability number (*B*_n_):^5^ at *B*_n_ > 1, microbial activity limits contaminant degradation, while at *B*_n_ < 1, mass transfer is the limiting process.^6−8^ One approach to overcome the bioavailability limitations of matrix-bound contaminants is to promote biodegradation through external electric field-induced contaminant release and transport (electro-bioremediation).^9^ Direct current (DC) electric fields induce various electrokinetic processes that allow for the targeted transport of bacteria and contaminants, including electrophoresis (EP), electromigration (EM), or the electroosmotic flow (EOF) in matrices.^10,11^ While EM and EP refer to the motion of charged entities toward electrodes of opposite charge, EOF reflects the surface charge-induced movement of pore fluids, typically flowing from the anode to the cathode.^12^ Contrary to parabolic pressure-driven hydraulic flow, the plug flow-shaped EOF acts at a nanometer distance above surfaces and allows for microscale mobilization of fluid fractions that are typically not affected by pressure-driven flow.^13,14^ It thus acts at scales relevant for contaminant–microbe–matrix interactions and promotes transport processes in inter- and intraparticle pore networks.^15−17^ Electrokinetic phenomena have been found to influence contaminant–sorbent interactions^18,19^ or the adhesion and transport of degrader bacteria.^20,21^ To date, electrokinetic remediation approaches have been proposed,^22,23^ for example, to (i) enhance the (surfactant-promoted) release of matrix-bound hydrophobic chemicals to improve their bioavailability for subsequent biotransformation, (ii) promote long-range mobilization of charged or highly water-soluble waterborne contaminants (electrokinetic mobilization), (iii) promote long-range transport and delivery of nutrients to microorganisms for enhanced contaminant biotransformation, or (iv) couple with advanced oxidation or reduction processes, ultrasound treatment, or with microbial contaminant degraders.^24−27^ Most approaches thereby have been applied for long-distance transport and removal (e.g., by electrokinetic permeable barriers) of charged or highly water-soluble contaminants from groundwater or the treatment of hydrophobic soil contaminants. For example, Niqui-Arroyo et al. showed the integration of bioremediation and electrokinetic flushing for enhanced PAH removal from creosote polluted soil.^28^ The electrokinetic treatment thereby promoted the intra-aggregate mobilization of poorly bioaccessible (i.e., slowly desorbing) PAH fractions in soil slurry phase bioremediation approaches either in the presence or the absence of nontoxic PAH-solubilizing surfactants. Here, we present a genuine different and new research aspect by quantifying the effect of the EOF on the biodegradation of a waterborne mobile chemical (naphthalene, NAH) by surface-attached bacteria in percolated biofiltration systems. Being dissolved in water at mg·L^–1^ concentrations, NAH is expected to be directly available to degrader cells attached to a nonsorbing surface that simultaneously serves as a porous material for the EOF. Although typically one to 2 orders of magnitude smaller than the pressure-driven bulk water flow in typical biofilter systems, the EOF creates a near-surface microscale fluid flow profile that is complementary to the bulk flow.^29,30^ By analogy with the use of EOFs for hydrodynamic ‘cloaking’ and ‘shielding’ of objects (such as glass beads) in a pressure-driven fluid flow,^31^ we here interrogate whether EOFs may enhance biodegradation by improving the microscale transport and availability of contaminants in the vicinity of surface-attached degradation cells.

To challenge our hypothesis, we assessed the effects of a weak DC electric field (*E* = 0.5 V·cm^–1^) on the biodegradation of dissolved NAH by *Pseudomonas fluorescens* LP6a in a laboratory biofilter system. Although NAH is readily degradable, it is often found in surface and stormwater.^32^ To change the NAH mass transfer, we used different NAH concentrations and varied the hydraulic loading in the range typically used in biofiltration systems. The specific NAH degradation rates (*q*_c_) in DC-free controls increased with increasing inflow concentrations but decreased with increasing Darcy velocities, suggesting bioavailability restrictions, depending on the hydraulic residence times. Relative to controls, DC promoted *q*_c_ and resulted in linearly increasing benefits relative to controls (DC benefits) from 0 to 55% with increasing hydraulic loadings. We interpret such DC benefits as EOF-enhanced NAH bioavailability. EOF allows for modified pressure-driven flow profiles around the collector beads at the microscale and therefore better NAH exposure to cells in zones of low NAH delivery.

## Materials and Methods

### Organism and Culture Conditions

NAH degrading^33^ and negatively charged^20^*Pseudomonas fluorescens* LP6a was cultivated in shaken 250 mL Erlenmeyer flasks with 100 mL of minimal medium (pH = 7)^34^ containing 1.5 g·L^–1^ NAH crystals as the sole carbon and energy source. The cultures were harvested in the early stationary phase, centrifuged at 3000 g for 10 min, and washed three times with a 100 mM potassium-phosphate buffer (PB; pH = 7.1, prepared by adding 0.061 mol K_2_HPO_4_ and 0.039 mol KH_2_PO_4_ in 1 L deionized water; ionic strength *I* = 0.22 M).^35^ The pellet was then resuspended in PB by vortexing to obtain an average optical density of OD_600 nm_ ≈ 0.037. A buffered electrolyte was applied to minimize electrolytically induced pH changes at the electrodes and to keep the pH in the columns between 7.2 and 7.5 as quantified by a pH meter (LAQUAtwin-pH-22, HORIBA, Japan).

### NAH Degradation Using Suspended Resting Cells

Dissolved NAH solutions were obtained by shaking excess amounts of crystalline NAH in PB in a light-protected Erlenmeyer flask on a rotary shaker (25 °C;150 rpm) for >48 h. Before use, residual crystals were removed by glass frit filtration. Biodegradation of dissolved NAH by suspended LP6a cells was studied under nongrowth conditions at room temperature. Strain LP6a was pregrown on NAH to OD_600 nm_ ≈ 0.66, the NAH crystals were then removed by filtration, and the cells were washed twice and finally resuspended in 20 mL 100 mM PB. Biodegradation was started by adding 1–2 mL of cell suspension to gently shaken saturated NAH solution (≈2.3 × 10^–4^ M) to obtain a final cell density of ≈7.7 × 10^7^ cells·mL^–1^ (OD_600 nm_ ≈ 0.051). One mL samples were taken every 5 min and acidified with a drop of concentrated sulfuric acid, and NAH concentrations were quantified by HPLC with UV detection at 250 nm (Shimadzu Class-VP) using an RP column (Nucleosil 100-5 C18 4 mm ID) and an isocratic MeOH/water (90:10 v/v) mixture at a flow of 1 mL·min^–1^ as eluent. Specific degradation rates (*q*_c_) were calculated, and the whole-cell Michaelis–Menten constant (*K*_s_) and the maximal degradation rates (*q*_max_) were derived using nonlinear parameter estimation.^36^ Abiotic NAH losses during the experiment in abiotic controls were <3%. NAH-containing solutions were handled with glass pipettes or glass syringes only.

### NAH Degradation in Percolation Columns

NAH biodegradation in the presence and absence (= control) of a DC electric field was quantified in vertical percolation columns (i.d.: 1 cm; l.: 10 cm) made of borosilicate glass as described earlier^20^ and sketched in Figure S1. The columns were sterilized prior to use and packed with clean, heat-sterilized (200 °C, 2 h) glass beads (diameter = 0.1–0.25 mm; Retsch, Germany). The packed bed had a porosity of ≈0.43 and a pore volume (PV) of 3.97 mL. Ensuring an average column to bead diameter ratio of >40 allowed for a representative elementary volume and to minimize sidewall flow.^37,38^ All columns were allowed to equilibrate by pumping clean PB for 30 min with a peristaltic pump from the top to the bottom. Two disk-shaped Ti/Ir electrodes (De Nora Deutschland GmbH, Germany) at the top (cathode) and bottom (anode) of the column were connected to a power pack (P333, Szczecin, Poland) that allowed the application of a constant DC electric field of *E* = 0.5 V·cm^–1^ and a current intensity of ≈3.6 mA. Due to the possible electrolytic formation of hydrogen (cathode) and oxygen (anode), the percolation system was constructed to minimize gas bubble transport through the columns (Figure S1).^21^ Both electrodes were placed at a distance to the reactive bed; the lid at the column top allowed the release of hydrogen, while an inclined glass frit separated the anode at the bottom and allowed for efficient removal of oxygen formed at the anode. The low-field strength prevented potential NAH electro-oxidation as inferred from sterile control columns and further approximated by cyclic voltammetry (Figure S2). The experiments were performed in four consecutive steps each (Figure S3) using variable flow rates (11.3, 19.3, 36.2, 50.6, and 59.6 mL·h^–1^; i.e., Darcy velocities of  = 0.2–1.2 × 10^–4^ m·s^–1^ and residence times of ≈4–21 min). In step 1, a well-stirred bacterial suspension (OD_600 nm_ = 0.03–0.04) was percolated through the columns for 3 PV; in step 2, the columns were then flushed with 3 PV of PB followed in step 3 by PB flushing in the absence (control) or presence of DC for 2 PV. The deposition of cells to the glass beads was determined by comparing the OD_600 nm_ of the bacterial suspensions in the influent (*C*_0_) and effluent (*C*). In step 4, an influent containing NAH concentrations *C*_0_ = 2.7, 5.1, or 7.8 × 10^–5^ mol·L^–1^ (i.e., 3.5, 6.5, or 10 mg·L^–1^) in PB containing nutrients (60% MM in 40% PB (200 mM) as electrolytes; *I* = 0.22 M) was percolated in the presence or absence of DC. NAH biodegradation was determined after each PV by comparing the NAH concentrations in the influent (*C*_0_) and effluent (*C*). One mL samples were taken, acidified, and measured by HPLC as described above.

### Determination of Cell Protein Content and Calculation of Surface Coverage

To determine the biomass development during NAH flushing, the protein content in the columns was quantified (i) at the beginning of NAH degradation (step 4), (ii) at 6 PV (DC) and 4 PV (no DC), when NAH in the respective effluents started to increase, (iii) at 8 PV (DC and no DC) when degradation had reached quasi-steady state, and (iv) at the end of the degradation experiments (Figure S3). At the given time points, all glass beads of a column were placed in a sterile-sealed 50 mL Erlenmeyer flask containing 5 mL of sterile PB. The column was rinsed three times with another 5 mL of sterile PB to ensure no residual glass beads remained in the column. The Erlenmeyer flask was then treated for 3 min in an ultrasonication bath (operating frequency: 35 kHz, Bandelin RK 255 H, Germany) and placed on a rotary shaker (25 °C; 150 rpm) for 1 h. After settlement of the glass beads, the supernatant (10 mL) was transferred to 15 mL Eppendorf tubes and centrifuged at 12000 g for 10 min and protein contents in the pellets quantified by the Bradford assay using bovine serum albumin as the protein standard.^39,40^ The relative surface coverage of the glass beads by LP6a cells based on the protein weights was calculated as described earlier.^21^

## Theory

### Description of NAH Biodegradation

Bacterial biodegradation of waterborne NAH can be described by the whole-cell Michaelis–Menten eq (eq 1).^41^ It describes the NAH flux through the cellular membrane *q*_c_ (nmol·mg^–1^_protein_·min^–1^) as a function of its concentration at the cell surface *C*_c_ (nmol·L^–1^), with *K*_s_ being the half-saturation concentration (nmol·L^–1^) resulting in *q*_c_*= q*_max_/2 with *q*_max_ being the maximal specific flux (nmol·mg_protein_ min^–1^).^42^
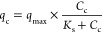
1

However, cellular NAH uptake reduces its concentration at the cell surface below the measured bulk concentration. *C*_c_ therefore depends on both the NAH uptake and the NAH transfer rate (*q*_t_) to the cell. For diffusive transport to cells (as to be expected in the vicinity of the cells in our system), *q*_t_ can be approximated by the product of the (biomass-related) mass transfer coefficient *k* (L·min^–1^·mg^–1^) and the concentration difference between the cell surfaces and the bulk NAH concentration (eq 2). Assuming similar *k* and microbial activity over the whole length of the column, *C*_c_ can be related to the NAH concentration in the inflow (*C*_0_).
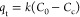
2

Assuming steady state (*q*_t_ = *q*_c_), eqs 1 and 2 can be combined to the so-called Best eq (eq 3).^42−44^ The Best equation allows us to correlate *q*_c_ to *k* without further information on *C*_c_, and hence to assess *k* in our columns in response to our experimental variations.
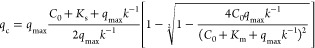
3

The NAH bioavailability can be estimated by the bioavailability number (*B*_n_).^27^*B*_n_ is the ratio of the mass transfer rate coefficient (*k*) to the microbial specific affinity *a*_A_^0^ = *q*_max_/*K*_s_. In analogy to this concept, we calculated the apparent *B*_n_ for NAH in our column (eq 4). *B*_n_ < 1 refers to a system that is likely controlled by mass transfer, while *B*_n_ > 1 refers to a system controlled by microbial activity.^5^
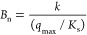
4

eq 5 refers to the DC benefit. It allows to quantify relative DC-induced promotion of NAH degradation in the glass beads-packed columns relative to DC-free controls:
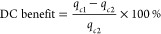
5with *q*_c1_ and *q*_c2_ being the NAH degradation rate at apparent steady-state outflow concentrations in the columns in the presence and absence of electric fields.

### Description of Hydraulic Flow Conditions and the Electro-osmotic Flow Velocity

The dimensionless Reynolds number (*R*_e_; eq S2)^45,46^ and the dimensionless Péclet number (*P*_e_, eq S4)^47^ were used to approximate the macroscopic flow conditions in our columns. *R*_e_ refers to the ratio of inertial forces to viscous forces within a fluid and allows to approximate if a flow is laminar (*R*_e_ < 1) or turbulent (*R*_e_ ≫1). *P*_e_ describes the relative importance of advection to diffusion over the characteristic length in our system that we assumed to be the average radius of the glass beads (*r*_bead_). The electroosmotic flow (EOF) velocity (*v*_EOF,r_, eq S5) in pores of the packed bed of our columns was approximated based on the simplified EOF expression of the Navier–Stokes equation as described in Supporting Information.

## Results

### Column Operating Conditions and Biomass Development

**Figure 1:**
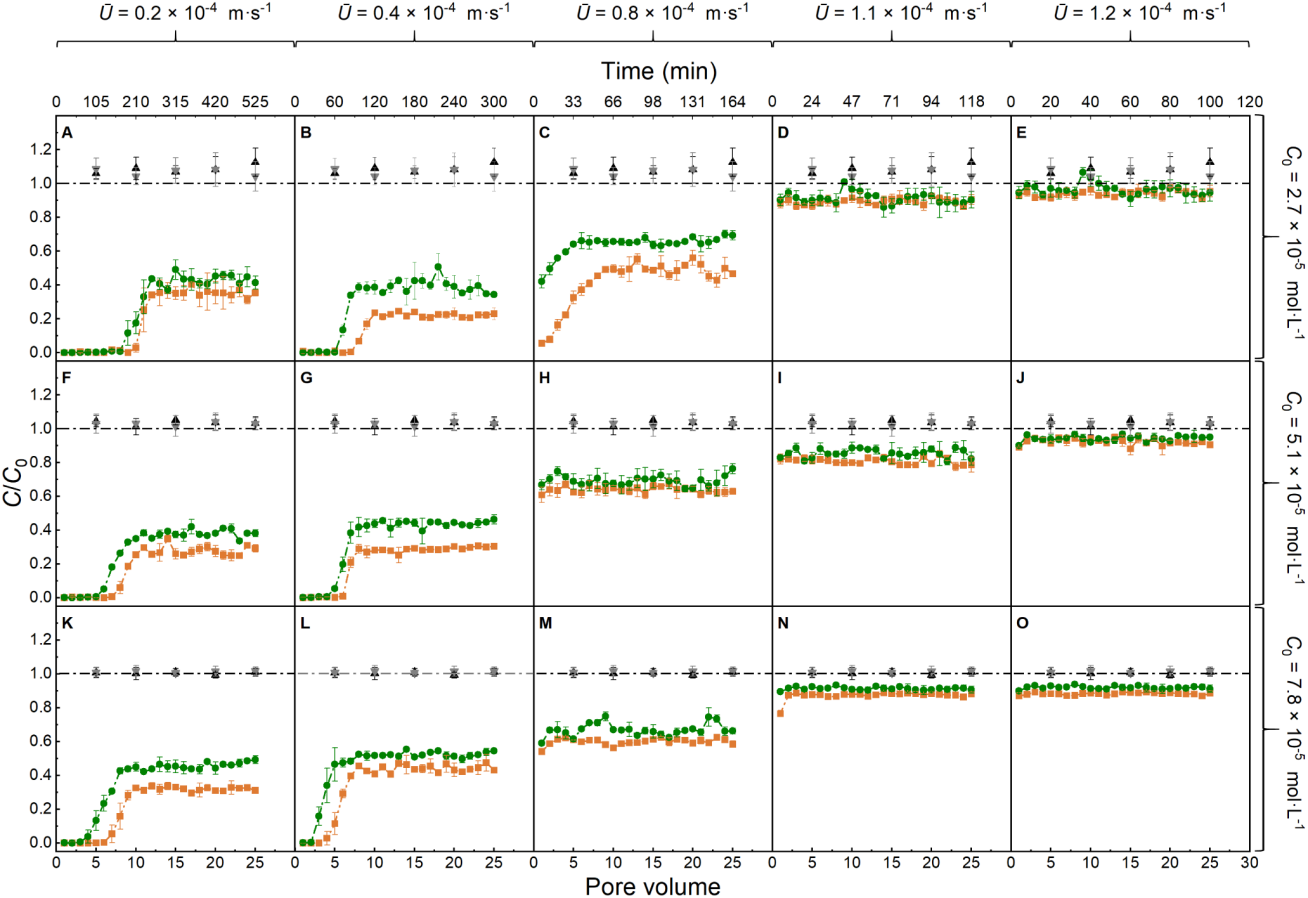
Relative NAH outflow concentrations after biodegradation by *P. fluorescens* LP6a in laboratory percolation columns. Relative NAH outflow concentrations in the absence (green) and presence of DC (*E* = 0.5 V·cm^–1^; orange). Light and dark gray triangles show relative outflow of cell-free columns in the absence and presence of DC (*E* = 0.5 V·cm^–1^). Columns depict the Darcy velocities ( × 10^–4^ = 0.2 m·s^–1^ (A, F, K), 0.4 m·s^–1^ (B, G, L), 0.76 m·s^–1^ (C, H, M), 1.1 (D, I, N), and −1.2 m·s^–1^ (E, J, O)). The rows reflect the three inflow NAH concentrations (*C*_0_ × 10^–5^ ≈ 2.7 mol·L^–1^ (A–E), 5.1 mol·L^–1^ (F–J), and 7.8 mol·L^–1^ (K–O)). The data represent averages and standard deviations of triplicate experiments. Relative outflow concentrations of columns with and without DC at apparent steady state were significantly different (*p* < 0.05) at all flow conditions and NAH concentrations.

NAH biodegradation in the laboratory biofilters was operated in the presence or absence of a low external DC electric field (*E* = 0.5 V·cm^–1^) at three NAH concentrations and five Darcy velocities ( = 0.2–1.2 × 10^–4^ m·s^–1^). Hydraulic loading corresponded to packed-bed residence times of 4–21 min and bulk laminar flow (*R*_e_ = 0.007–0.04 < 1) with advective transport being 6–34 times more important than diffusion (*P*_e_ = 6–34; Table S1). Biomass development in the columns was quantified by time-resolved protein measurements (Figure S3, Table S2). Initial biomass was independent of the hydraulic flow rates used for loading and was ≈8% lower in the presence of DC (≈24 ± 1.3 μg_protein_) than in DC-free controls (≈27 ± 1.3 μg_protein_, Table S2). This was likely due to the electrokinetic removal of loosely attached cells during flushing^48^ (step 3 in Figure S3). During NAH loading (step 4, Figures 1 and S3), biomass developed depending on NAH concentrations, flow conditions, and the presence of DC (Table S2). At the two lower hydraulic loadings ( = 0.2 and 0.4 × 10^–4^ m·s^–1^), the biomass increased to ≈36–59 μg_protein_ (control) and ≈40–72 μg_protein_ (DC), while slight or no change was observed at intermediate ( = 0.8 × 10^–4^ m·s^–1^) and highest flow rates ( = 1.1 and 1.2 × 10^–4^ m·s^–1^) independent of DC presence (Table S2). Biomass increased during the first 2–12 PV after NAH flushing and then remained quasi-constant under both control and DC conditions. Assuming a homogeneous distribution of the biomass in the columns, the cells were calculated to cover 0.1–0.2% of the glass bead surfaces only (eq S1, Table S3).

### NAH Biodegradation in the Absence of DC

**Figure 2:**
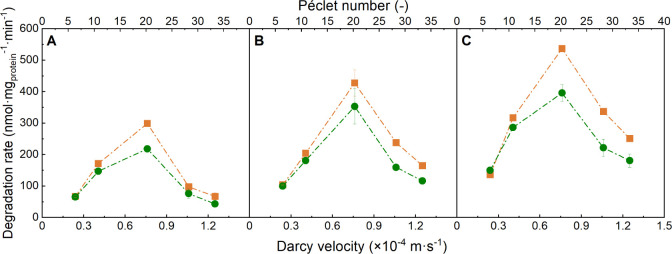
NAH degradation rates at different NAH inflow concentrations, Darcy velocities, and corresponding Péclet numbers in the absence (green) and presence of DC (orange, *E* = 0.5 V·cm^–1^). A: *C*_0_ ≈ 2.7 × 10^–5^ mol·L^–1^. B: 5.1 × 10^–5^ mol·L^–1^. C: 7.8 × 10^–5^ mol·L^–1^.

NAH biodegradation by *P. fluorescens* LP6a was inferred from NAH breakthrough curves applying NAH inflow concentrations (*C*_0_ = 2.7–7.8 × 10^–5^ mol·L^–1^) at *C*_0_ > *K*_s_. The Michaelis–Menten parameters for NAH degradation by strain LP6a were estimated from separate batch experiments at nongrowth conditions with *K*_s_ = 2.5 × 10^–6^ mol·L^–1^ and *q*_max_ = 555 nmol·mg_protein_^–1^·min^–1^ and a specific affinity a_A_^0^ = 0.22 L· mg_protein_^–1^ min^–1^. At lower flow rates, we observed no NAH in the outflow in the first 1–8 PV. NAH concentrations then increased and typically reached a steady value (plateau) at 5–8 PV with outflow concentrations increasing with hydraulic loadings and *C*_0_ (Figure 1). At high-flow velocities and/or highest *C*_0_, by contrast, high relative outflow concentrations were observed already from the first PV. Specific NAH biodegradation rates *q*_c_ were determined at stable NAH outflow (Figures 2A–C). They ranged from 43 to 396 nmol·mg_protein_^–1^·min^–1^ and were consistently lower than *q*_max_ in well-stirred batch experiments. At a given hydraulic loading, *q*_c_ increased with increasing *C*_0_ (Figures 2A–C), while at a given *C*_0_, the degradation rate *q*_c_ first increased with higher flow rates and then sharply decreased at  > 0.8 × 10^–4^ m·s^–1^. For example, at *C*_0_ = 2.7 × 10^–5^ mol·L^–1^, *q*_c_ increased from 65 to 218 nmol·mg_protein_^–1^·min^–1^ and subsequently decreased to 43 nmol·mg_protein_^–1^·min^–1^ at  ≥ 0.8 × 10^–4^ m·s^–1^ (Figure 2A).

### NAH Biodegradation in the Presence of DC

Applying a DC field of *E* = 0.5 V·cm^–1^ resulted in improved biodegradation, as evidenced by significantly reduced NAH outflow concentrations. At the two lower flow rates and lowest *C*_0_, DC led to extended periods of complete NAH degradation (Figure 1) but to otherwise similar shapes of the NAH breakthrough curves as in the controls. No NAH removal was observed in cell-free columns (Figure 1) or columns loaded with heat-inactivated bacteria (Figure S4) indicating the absence of electrochemical degradation or significant NAH sorption to biomass. The absence of NAH electrooxidation at *E* = 0.5 V·cm^–1^ was further verified by cyclic voltammetry (Figure S2). Applying a DC field consistently led to 4–55% higher *q*_c_ than in DC-free columns at ranges from 67 to 537 nmol·mg_protein_^–1^·min^–1^ (Figures 2A–C). The DC benefits were independent of the NAH concentration, but they increased by ≈0–6%, 10–17%, 30–37%, 39–55%, and 41–52% with increasing flow rates.

## Discussion

### NAH Biodegradation Rates Vary in Response to Hydraulic Flow Regimes

We analyzed the effects of a weak DC electric field on the biodegradation of waterborne NAH in packed-bed laboratory percolation systems at NAH concentration *C*_0_ ≫ *K*_s_. NAH was used as a readily degradable environmental contaminant and *P. fluorescens* LP6a as a well-described NAH degrader^49^ exhibiting a specific affinity typical for NAH degrading bacteria.^50−52^ To change NAH availability to the LP6a cells, we varied NAH concentrations and also altered the hydraulic loadings to cover typical hydraulic residence times reported for biofiltration systems (2–120 min.^1^ By using NAH concentrations >10 K_s_, we were able to maintain sufficient bulk NAH concentrations to allow maximum intrinsic NAH transformation by *P. fluorescens* LP6a over the entire column length, even at low NAH effluent concentrations. During NAH loading, biomass increased 2–3-fold at initial PV depending on NAH concentrations and residence times (Table S2); however, high-flow conditions did not provide sufficient NAH fluxes to cells to promote LP6a growth but only allowed to maintain constant microbial activity. Since only 0.1–0.2% of the glass bead surface was covered by bacteria (Table S3), it is improbable that interbacterial shielding and/or competition effects would restrict NAH availability to individual cells.

The specific NAH biodegradation rates increased with increasing inflow NAH concentration but showed opposing trends in response to increasing Darcy velocities at a given *C*_0_. While biodegradation rates increased at increasing Darcy velocity up to  > 0.8 × 10^–4^ m·s^–1^, they again decreased at higher flow rates. Such an observation is in line with the literature describing contaminant biodegradation in porous media at comparable flow conditions under both growth and nongrowth conditions and was explained by the dependency of biodegradation rates on the mean contact times (i.e., hydraulic retention time) between a contaminant and bacteria due to microbial substrate uptake restrictions.^53−56^ While the time required for diffusive NAH transfer and uptake by cells is independent of the flow velocity, the time needed for advective transport is inversely proportional to the hydraulic flow velocity in packed beds.^7,57,58^ Therefore, *P*_e_ (i.e., the ratio of the time scale needed for diffusive and convective transport over a given transport length) seems a good descriptor for NAH biodegradation in our system (Figure 2A-C). We hence further tested the influence of bead size (as a driver of the transport length; cf., eq S4) at constant hydraulic flow and *C*_0_ and found a good linear correlation of *q*_c_ and *P*_e_ (Figure S5). At fixed hydraulic flow, increasing the inflow of NAH concentrations *C*_0_ led to increased degradation rates. We interpret this as an indication for transport-limited NAH availability to cells in our system. As *C*_0_ ≫ *K*_s_, NAH degradation otherwise would not increase with increasing concentration assuming whole-cell Michaelis–Menten kinetics.

### Bioavailability Numbers Point at Uneven Microscale Flow Profiles and Biomass Distribution

**Figure 3:**
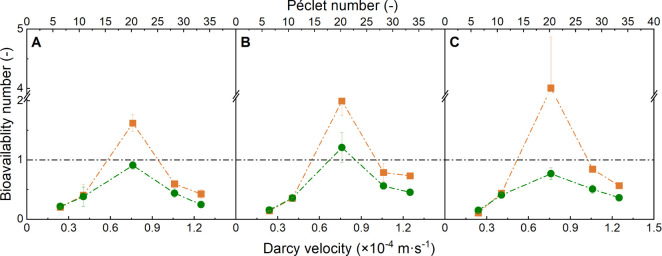
Effects of the Darcy velocity and concomitant Péclet numbers on calculated NAH bioavailability numbers (*B*_n_) calculated for the absence (green) and presence of DC (orange, *E* = 0.5 V·cm^–1^) at given NAH inflow concentrations. A: *C*_0_ ≈ 2.7 × 10^–5^ mol·L^–1^. B: 5.1 × 10^–5^ mol·L^–1^. C: 7.8 × 10^–5^ mol·L^–1^.

To estimate NAH bioavailability to the degrading biomass in our columns, we derived the (biomass-related) mass transfer coefficients (*k*) using the so-called Best eq (eq 3) as input for the bioavailability number of our system. *B*_n_ < 1 refers to NAH biodegradation that is controlled by mass transfer, while *B*_n_ > 1 reflects columns that are controlled by microbial activity.^5^ As *B*_n_ was derived from *q*_c_, it also showed similar hydraulic flow-dependent trends as *q*_c_. This means that *B*_n_ confirmed low NAH availability to degraders at higher flow rates, regardless of *C*_0_ (Figure 3). At intermediate hydraulic loading, however, *B*_n_ reached unity despite *q*_c_ < *q*_max_. This indicates that only a fraction of the total measured biomass in our column was actively degrading NAH, that is, contributed to the apparent *k* calculated for our systems. Since the relative bacterial outflow concentrations during loading reached almost unity at all flows (i.e., all collectors in the packed bed were equally exposed to bacteria; Figure S3, steps 1 and 2), we exclude nonhomogeneous biomass distribution along the flow path of the columns as the reason. We rather propose uneven bacterial biomass distribution on the individual glass beads. Irregular loading to single collectors may have resulted from uneven transport velocities along beads and concomitantly reduced deposition probability of bacteria (“shadow zones”) induced by a shear component of the tangent flow around collector beads.^58^ Uneven fluid flow velocity along the bead surfaces may additionally hinder the transfer of NAH to degrader cells in a packed bed. At laminar flow, the flow velocity around spherical collectors depends on the location of their surface. For instance, low flow at the front and rear stagnation points may prevail (Figure 5) likely leading to reduced shadow effects and locally higher biomass loading.^59^ Therefore, an emerging interplay between uneven flow and uneven biomass distribution at the microscale may result in the reduced NAH availability and biodegradation of LP6a cells at increasing flow rates.

### EOF Changes Microscale Flow Profiles and Increases NAH Biodegradation during Biofiltration

**Figure 4:**
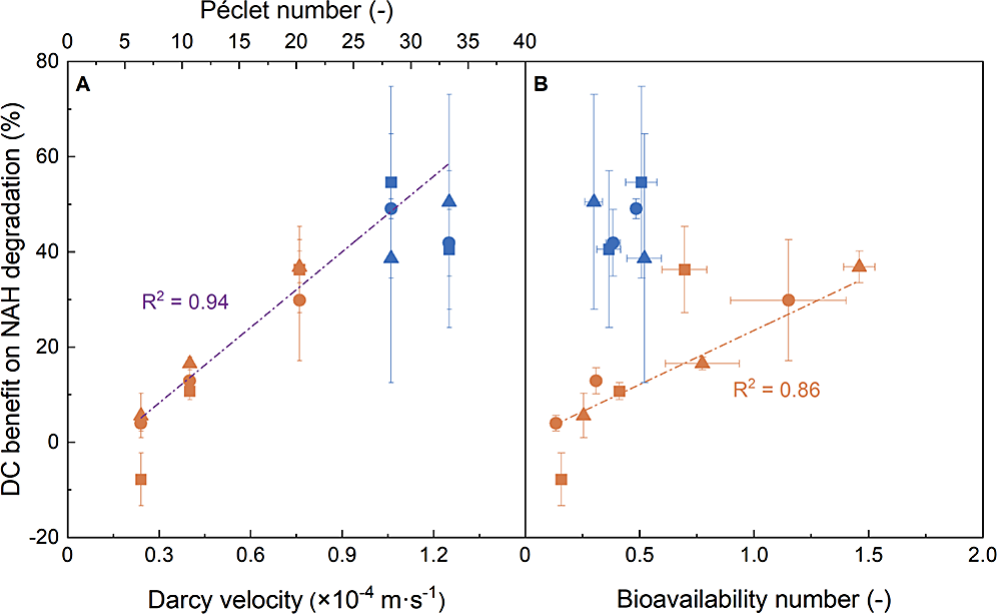
Effects of Darcy velocities (A) and bioavailability numbers (*B*_n_; B) on relative DC benefit on NAH biodegradation by *P. fluorescens* LP6a. Triangles, circles, and squares represent benefit for *C*_0_ ≈ 2.7 × 10^–5^ mol·L^–1^, 5.1 × 10^–5^ mol·L^–1^, and 7.8 × 10^–5^ mol·L^–1^, respectively. Blue symbols and orange symbols in panel B highlight *B*_n_ calculated for high-flow conditions (*U* > 0.8 × 10^–4^ m·s^–1^) and low-flow conditions (*U* < 0.8 × 10^–4^ m·s^–1^). Data represent averages and standard deviations of triplicate experiments.

Applying an EOF opposite to the hydraulic loading resulted in significantly reduced NAH breakthrough concentrations and 4–55% increased removal rates (Figure 4A). The DC benefits increased linearly with flow rates and associated Péclet numbers (Figure 4A). The observed NAH removal is to be attributed to microbial degradation, as no electrooxidation and insignificant NAH sorption to the (very low) biomass (22–61 μg_protein_) in the column were observed (Figures S3 and S4). Higher biodegradation rates and equal or higher biomass development than in DC-free controls also suggest that the weak DC field had no negative impact on the physiology of the biocatalysts as was observed earlier in other studies on DC field effects on contaminant-degrading bacteria.^60,61^ We used a buffered electrolyte to avoid electrolytically induced pH changes that could potentially affect microbial physiology or lead to changed EOF and thereby mask initial DC field effects. Our data indicate that DC fields promote NAH bioavailability and biodegradation at increasing Péclet numbers (Figure 4), that is, in situations of increasing ratios of time scales needed for diffusive and convective transport, respectively over a given length. To further challenge such findings, we changed the size of glass beads but not the hydraulic loading to vary *P*_e_ (Figure S5). We likewise found a high correlation between DC benefit and *P*_e_ pointing at a DC-related reduction of apparent diffusion times for NAH degradation. As the EOF is a surface-associated water movement taking place at a nanometer distance from the bead surface, EOF reduces the effective thickness of the diffusive boundary layer above the surface of the glass beads and attached bacteria, respectively. It may thus allow for a reduction in the time scales required for the diffusive transport of NAH through the diffusive boundary layer to the bacteria^14^ without changing the overall hydraulic flow of the bulk liquid that was ≈100–800 times higher than the calculated near surface EOF (3.4 × 10^–7^ m·s^–1^).

**Figure 5:**
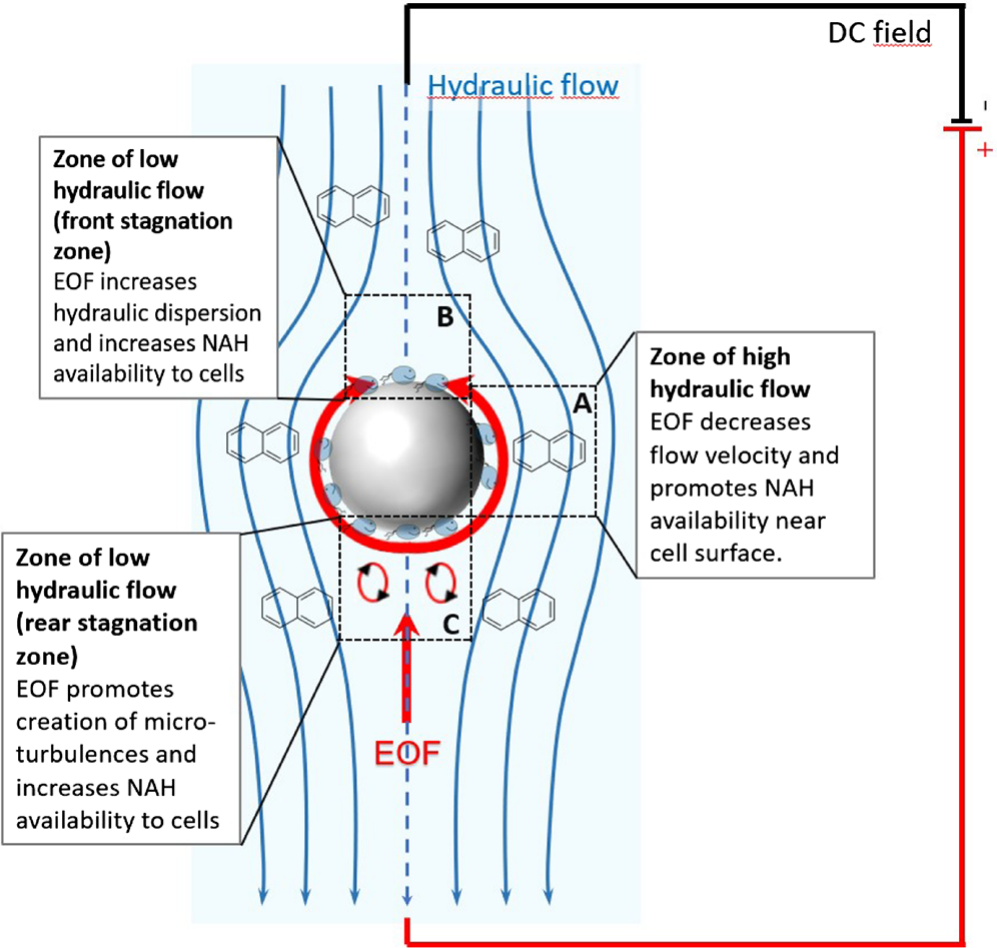
Proposed mechanisms of DC field-induced EOF effects on improved NAH biodegradation.

Comparing the DC benefit to bioavailability numbers for corresponding DC-free scenarios, we found good collinearity at lower flow rates, that is, poor DC benefits at *B*_n_ ≪ 1 and high effects at *B*_n_ ≈ 1, respectively. The highest DC benefit, however, was seen at both the highest Darcy velocity, where unsuitable flow conditions led to *B*_n_ ≈ 0.5 and *q*_c_ < *q*_max_ (Figure 4B), respectively. Here, electrokinetic effects seem to have effectively increased the NAH transport to the degrading biomass in the column. As shown by Omar et al., EOF in porous materials creates complex flow fields that deviate from pure pressure-driven hydrodynamics by promoting inhomogeneous flow and microscale vortices in flow shadow zones.^30^ EOF impact on net liquid flow at the microscale is largely influenced by the orientation of the pore throats and EOF-creating surfaces relative to the direction of the applied electric field. For instance, electroosmotic slips are expected to be higher along surfaces and pore structures that are aligned along the electric field. If pressure-driven and electroosmotic flows are counteracting, lowered dispersion may prevail and so locally increase NAH retention and concomitant NAH exposure times and NAH availability to degrading bacteria.^31^ In our system, these are likely the points of the highest flow velocity above the glass bead surfaces (zone A; Figure 5). However, in situations, where pressure and electrical drive only partially counteract, higher dispersion (zone B; Figure 5) or the creation of microscale turbulences (“eddies”, zone C; Figure 5) and subsequent higher NAH mass transfer and availability to cells may be expected near the bead surfaces. Both factors, that is, the increase of local residence times at points of high flow and increased EOF-driven mass transfer to cells in zones of low fluid flow, may explain the observed DC benefits on NAH bioavailability and biodegradation. The relative importance of both mechanisms will dynamically depend on the distribution and development of the active biomass. Despite such emerging complex interplay, we found a good correlation between the DC benefits and *P*_e_, suggesting the macroscopically derived *P*_e_ to be a good indicator for the electric field effects at varying running conditions. Future studies should investigate the effects of the relative directions of the EOF and the hydraulic flow or the application of electric fields of varying polarity to avoid possible negative pH effects in biofiltration systems. Assuming a similar biomass distribution on the glass beads and aligned EOF and pressure-driven flow, we would expect similar or slightly reduced DC benefits for biodegradation. Similar to opposing flow directions, the EOF would result in increased mixing in the front and rear shadow zones of the beads (zone C; Figure 5), whereas the effect of the EOF on NAH bioavailability in zone A (Figure 5) remains elusive. Here, aligned EOF and hydraulic flow may reduce the residence time of NAH but still increase NAH delivery to the cells due to its effect on the diffusive boundary layer of the cell surfaces. Such an assumption is supported by preliminary experiments in our system ( = 0.8 × 10^–4^ m·s^–1^ and *C*_0_ = 7.8 × 10^–5^ mol·L^–1^) revealing benefits of 50 ± 12% and 26 ± 2% for opposing and aligned flow conditions, respectively. Detailed further analyses however will be required to corroborate our findings and assumptions by, for example, means of microscale research of fluid flow patterns and bacterial biomass development in the presence and absence of electrokinetic effects. This may be achieved by combining experiments in microfluidic devices with modeling approaches.^30^

### Relevance for Environmental and Biotechnological Applications

Up to date, most electrokinetic contaminant removal approaches have been used for the release and subsequent biotransformation (electro-bioremediation) of soil-bound chemicals or the DC-field-driven promoted long-distance transport of waterborne chemicals (electrokinetic mobilization).^14,62^ Here, we quantified the effect of the EOF on the biodegradation of a readily water-soluble, waterborne chemical by surface-attached bacteria in a biofiltration system under varying hydraulic conditions. Despite the low DC electric field applied and negligible effects of EOF on the bulk water flow, our data show a DC benefit up to 55% for NAH biodegradation, with benefits being independent of NAH inflow concentrations. Observing that NAH bioavailability depended on pressure-driven hydraulic loading, we mechanistically explain that electroosmosis may alter the fluid flow profile around the glass beads, thereby promoting NAH mass transfer to the attached cells, for example, in water flow shadows around the beads. Therefore, our results give rise to further studies combining small-scale laboratory experiments (e.g., in microfluidic devices) and computational fluid dynamics. Such investigations at microscale will allow us to challenge our mechanistic interpretation of the data presented.

Our findings may simultaneously give rise to novel biofilter systems or other applications that require, for example, maintained biodegradation at fluctuating hydraulic loads. In biofiltration, the residence time (or empty bed contact time, EBCT) is one of the most important operational parameters. For instance, in drinking water treatment, an EBCT of 2–20 min is recommended, while for wastewater treatment, EBCTs of 20–120 min may be employed.^3^ Further application may be possible in biofilter systems combining the temporal retention of contaminants by targeted sorbents with biodegradation. Previous studies have shown that both, chemical sorption and bacterial deposition, can be influenced by DC electric fields.^20^ Such an application may be thought of, for example, the treatment of storm- or street runoff water.^2^ However, a further mechanistic understanding will be needed to fully profit from the effects of electrokinetic phenomena in porous media. For instance, the effects of DC fields and their strengths on contaminants on biodegradation of emerging contaminants at low or trace concentrations and/or complex contaminant mixtures should be further studied. Likewise, EOF-induced hydraulic pressure profiles and their effects on biodegradation sorption and (bio)colloid deposition may vary depending on the characteristics of the packed bed, the metabolic activity and diversity of the biomass, and/or the environmental conditions.

## Supporting Information Available

Calculation of the bacterial surface coverage of glass beads; procedure for bacterial inactivation; description of macroscopic flow conditions; description of the electroosmotic flow velocity; overview of flow conditions applied, relevant hydraulic parameters, and estimated electro-osmotic flow velocity at *E* = 0.5 V·cm^–1^; protein content development of *P. fluorescens* LP6a during NAH degradation at variable conditions; calculated surface coverage of glass beads by *P. fluorescens* LP6a cells during NAH degradation at variable experimental conditions; schematic view of the percolation column setup; cyclic voltammograms of NAH during electrolysis under different conditions; representatively relative outflow concentrations of *P. fluorescens* LP6a cells and NAH in the absence and presence of DC at *E* = 0.5 V·cm^–1^; relative NAH outflow concentrations in columns with heat-inactivated *P. fluorescens* LP6a cells in the absence and presence of DC at *E* = 0.5 V·cm^–1^; effect of glass bead diameter-dependent Péclet numbers on NAH degradation rates and DC-induced degradation benefits in absence and presence of DC at *E* = 0.5 V·cm^–1^ (PDF)
