# Supplementary material for: DC Electric Fields Promote Biodegradation of Waterborne Naphthalene in Biofilter Systems
Source: Environ Sci Technol. 2024 Oct 1;58(41):18234–43. doi: 10.1021/acs.est.4c02924 (PMC11483754; doi:10.1021/acs.est.4c02924)
Supplement: Supplementary file 1 — es4c02924_si_001.pdf [file es4c02924_si_001.pdf]

SUPPLEMENTARY INFORMATION

DC Electric Fields Promote Biodegradation of Waterborne Naphthalene in Biofilter Systems

Jinyao He[1], Jose Carlos Castillo-Alcantara[1,2#], Jose Julio Ortega-Calvo[2], Hauke Harms[1] and Lukas Y. Wick[1]*

1. *Department of Applied Microbial Ecology, Helmholtz Centre for Environmental Research UFZ, 04318 Leipzig, Germany.*
2. *Instituto de Recursos Naturales y Agrobiología de Sevilla (IRNAS-CSIC), Avda. Reina Mercedes 10, E-41012 Seville, Spain.*

[#] *present address: International Research Center in Critical Raw Materials for Advanced Industrial Technologies (ICCRAM), University of Burgos, Centro de I+D+I. Plaza Misael Bañuelos s/n. 09001, Burgos, Spain*

This supplementary information contains:

- Information on 'Materials and Methods' and 'Theory'
- 3 Tables
- 5 Figures
- References

[*] Corresponding author: Mailing address: Helmholtz Centre for Environmental Research – UFZ. Department of Applied Microbial Ecology, Permoserstraße 15, 04318 Leipzig, Germany. phone: +49 341 6025 1316, e-mail: lukas.wick@ufz.de.

MATERIALS AND METHODS

Calculation of surface coverage

Assuming that the entire glass surface allows for irreversible adhesion, the fraction (%) of bacterial coverage on the surface can be described by eq. S1

$$\theta\ (\%) = \frac{N_{\mathrm{b}}\pi r_{\mathrm{bacteria}}^2}{N_{\mathrm{s}}4\pi r_{\mathrm{bead}}^2} \times 100 \qquad \text{(S1)}$$

where N_s and N_b are the numbers of glass bead and bacteria in the column and r_{bead} and $r_{bacteria}$ the radii of bacteria and the glass beads, respectively.[1] N_s and N_b can be estimated by the non-porous volume of the collectors and the measured protein content, respectively.

Inactivation of bacteria

For thermal inactivation of bacteria, cultured *strain* LP6a was precipitated by centrifugation in 3000 xg for 10min. After discharging the supernatant, the pellet was washed three times with a 5mL sterile PB. Finally, the pellet was resuspended in 20mL PB. Inactivation step was applied by placing the obtained cell suspensions in the water bath at 70°C for 40min. The heating-treated cell suspensions were prepared for column experiments. To ensure that all of the bacterial cells were killed, a culture of bacteria in LB media was provided and incubated overnight.[2]

THEORY

Description of macroscopic flow conditions

The dimensionless Reynolds number (R_e; eq. S2)[3-4] was used to approximate fluid flow patterns in our percolation columns that were filled with glass beads. The R_e reflects the ratio of inertial forces to viscous forces within a fluid and allows to approximate if a flow is laminar or turbulent.

$$R_{\mathrm{e}} = \frac{d\overline{U}\rho_{\mathrm{f}}}{\eta(1-n)} \qquad \text{(S2)}$$

The d (1.75 x 10^{-4} m) is the diameter of the spherical glass beads, ρ_f (998 kg·m^3) is the fluidic density, η (0.001 Pa·s) is kinematic viscosity and n (0.43) is the porosity. If $R_e < 1$, i.e. laminar flow prevails, the superficial fluid velocity (Darcy velocity ($\overline{U}$), m·s^{-1}) can be calculated by eq. S3, where A is the cross-sectional area (1.33 x 10^{-4} m^2), Q (mL·h^{-1}) the volumetric flow rate, and $\overline{V}$ (m·s^{-1}) the pore fluid velocity

$$\overline{U} = n\overline{V} = Q/\mathrm{A} \qquad \text{(S3)}$$

The dimensionless Péclet number (P_e)[5] was used to approximate the relative importance of advection to diffusion in our system (eq. S4). The P_e is defined as the ratio of advective rate to diffusive rate, and hence combines macroscale and microscale processes of laminar flow through the porous media.[6] The P_e hence is the ratio of time scales required for diffusive (D_{dif} is the diffusion coefficient of NAH, m^2·s^{-1}) and convective transport over a characteristic length r_{bead}

$$P_e = \frac{\overline{V} r_{\mathrm{bead}}}{D_{\mathrm{dif}}} \quad \text{(S4)}$$

Description of the electroosmotic flow velocity

The electroosmotic flow velocity in an intra-particle pore of radius *r* (nm) from the collector (i.e. the glass bead) $v_{\mathrm{EOF,r}}$ can be derived from the simplified EOF expression of the Navier-Stokes equation (eq. S5) based on the maximal v_{EOF} ($v_{\mathrm{EOF,max}}$, eq. S6) and the α*r* function[7] (eq. S7) that refers to the characteristics of the porous medium where the EOF is taking place

$$V_{\mathrm{EOF,r}} = -\frac{\varepsilon_0 \varepsilon_{\mathrm{r}} \zeta_{\mathrm{C}} n \tau \times E}{\eta} \times \left(1 - \frac{2 f_1(ar)}{ar f_0(ar)}\right) \quad \text{(S5)}$$

$$V_{\mathrm{EOF,max}} = -\frac{\varepsilon_0 \varepsilon_{\mathrm{r}} \zeta_C n \tau \times E}{\eta} \quad \text{(S6)}$$

$$f(\alpha r) = 1 - \frac{2 f_1(\alpha r)}{\alpha r f_0(\alpha r)} \quad \text{(S7)}$$

$$\alpha^{-1} = \left[3.29 z \sqrt{C_{\mathrm{m}}}\right]^{-1} \quad \text{(S8)}$$

ε_r is the dielectric constant of water (78.5), ε_0 (8.85×10^{-12} F·m^{-1}) is the vacuum permittivity, ζ_C (mV) is the zeta potential of the collector surface at the experimental conditions, τ refers to the tortuosity (1.8) of the glass bead,[8] and *E* (V·cm^{-1}) is the electric field strength applied. Here f_0 and f_1 are the zero and first-order modified Bessel functions,[7] the term αr reflects the ratio of the pore radius ($r = (\sqrt{2} - 1) \times r_{\mathrm{bead}}$) to the thickness of the double layer. α^{-1} is the thickness of the electrical double layer (EDL, nm) calculated by the Guoy-Chapman theory[9] with C_m (mM) and *z* being the molar bulk concentration and the charge number of electrolytes.[10] For a 100 mM PB with nutrients, a α^{-1} of 0.65 nm was calculated.

Table S1. Overview of flow conditions, Reynolds and Péclet numbers and estimated electro-osmotic flow velocity at *E*= 0.5 V·cm^{-1} in the percolation columns: Darcy velocity ($\overline{U}$, eq. S3) and pore velocity ($\overline{V}$, eq. S3), the electro-osmotic flow velocity ($v_{EOF,r}$, eq. S5), Reynolds number (R_e, eq. S2), Péclet number (P_e, eq. S4), and Π *as* the ratio of $\overline{V}$ and $v_{EOF,r}$

Flow rate	Darcy velocity	Pore velocity	Electro-osmotic flow velocity	Ratio	Reynolds number	Péclet number
(mL·h^{-1})	$\overline{U}$ (×10^{-4} m·s^{-1})	$\overline{V}$ (×10^{-4} m·s^{-1})	$v_{EOF,r}$ (× 10^{-7} m·s^{-1})	$\Pi = \overline{V} / v_{EOF,r}$ (× 10^{2})	R_e (× 10^{-2})	P_e
11.3	0.2	0.6	3.4	1.8	0.7	6
19.3	0.4	0.9	3.4	2.6	1.2	11
36.2	0.8	1.8	3.4	5.3	2.3	21
50.6	1.1	2.5	3.4	7.4	3.3	29
59.6	1.2	2.9	3.4	8.5	3.8	34

Table S2. Protein content development of *P. fluorescens* LP6a during NAH degradation in percolation columns at different flow regimes, NAH concentrations in presence and absence of DC (E = 0.5 V·cm^{-1}). The data represent average and standard deviations (brackets) of n = 3. The time points (i, ii, iii, iv) reflect protein content after loading (i), at point of increasing NAH outflow concentrations (ii), at begin of stable NAPH outflow concentration (iii), and at the end of the experiment (iv) as exemplified in Fig. S3.

Darcy velocity $\overline{U}$	Bulk conc. C_b	Protein loaded to columns (i)		Protein at the end of zero NAH outflow (ii)		Protein at begin of stable NAH outflow (iii)		Protein at the end of experiment (iv)	
		(DC)	(no DC)	(DC)	(no DC)	(DC)	(no DC)	(DC)	(No DC)
(m·s^{-1})	(mg·L^{-1})	(µg)							
0.2 × 10^{-4}	3.5	23.0 (0.4)	25.0 (0.6)	51.2 (0.4)	46.2 (0.6)	52.4 (0.4)	47.6 (0.6)	52.4 (0.4)	47.7 (0.6)
	6.5	26.5 (0.3)	27.9 (0.3)	56.8 (0.3)	49.2 (0.3)	65.2 (0.3)	58.4 (0.3)	65.5 (0.2)	58.6 (0.3)
	10	25.6 (0.6)	27.2 (1.4)	60.7 (0.6)	44.6 (1.7)	71.7 (0.7)	55.5 (2.3)	71.9 (0.6)	55.6 (2.3)
0.4 × 10^{-4}	3.5	23.7 (0.3)	25.5 (0.5)	37.9 (0.3)	35.4 (0.5)	39.8 (0.3)	36.3 (0.7)	39.8 (0.2)	36.4 (0.7)
	6.5	25.3 (0.5)	26.7 (0.7)	45.5 (0.2)	40.3 (0.6)	56.7 (0.2)	46.4 (0.4)	56.9 (0.1)	46.6 (0.5)
	10	23.1 (0.2)	25.4 (0.3)	34.0 (0.2)	32.7 (0.3)	43.1 (0.2)	41.8 (0.3)	43.2 (0.3)	41.8 (0.3)
0.8 × 10^{-4}	3.5	25.4 (0.4)	27.1 (0.4)	-	-	31.8 (0.4)	29.4 (0.5)	31.9 (0.4)	29.5 (0.6)
	6.5	23.1 (0.3)	24.9 (0.3)	-	-	24.6 (0.3)	25.7 (0.3)	24.7 (0.3)	25.9 (0.3)
	10	25.1 (0.2)	27.6 (2.0)	-	-	32.5 (0.2)	35.5 (2.0)	32.6 (0.2)	35.6 (2.0)
1.1 × 10^{-4}	3.5	25.6 (0.5)	27.2 (0.4)	-	-	25.8 (0.5)	27.3 (0.4)	25.8 (0.5)	27.4 (0.4)
	6.5	23.7 (0.2)	25.7 (0.2)	-	-	24.2 (0.2)	26.1 (0.2)	24.3 (0.2)	26.1 (0.2)
	10	22.7 (0.2)	25.2 (0.2)	-	-	24.0 (0.2)	25.7 (0.3)	24.3 (0.2)	25.7 (0.3)
1.2 × 10^{-4}	3.5	25.2 (0.2)	26.9 (0.4)	-	-	25.4 (0.2)	27.0 (0.4)	25.4 (0.2)	27.1 (0.4)
	6.5	21.8 (0.3)	23.4 (0.3)	-	-	22.4 (0.4)	23.8 (0.2)	22.4 (0.4)	23.8 (0.2)
	10	25.3 (0.3)	28.4 (0.9)	-	-	25.8 (0.4)	28.9 (0.9)	25.9 (0.4)	29.0 (0.8)

Table S3. Estimated surface coverage of glass beads by *P. fluorescens* LP6a cells during NAH degradation in percolation columns at different flow regimes, NAH concentrations and time points in presence and absence of DC (E = 0.5 V·cm^{-1}). The data represent average and standard deviations (brackets) of n = 3. The time points (i, ii, iii, iv) reflect protein content after loading (i), at point of increasing NAH outflow concentrations (ii), at begin of stable NAH outflow concentration (iii), and at the end of the experiment (iv) as exemplified in Fig. S3.

**Darcy velocity** $\overline{U}$	**Bulk conc.** C_b	**Initial surface coverage (i)**		**coverage at the end of zero NAH outflow (ii)**		**coverage at begin of stable NAH outflow (iii)**		**coverage at the end of experiment (iv)**	
		(DC)	(no DC)	(DC)	(no DC)	(DC)	(no DC)	(DC)	(no DC)
(m·s^{-1})	(mg·L^{-1})	(× 10^{-2}, %)							
0.2 × 10^{-4}	3.5	8.0 (0.1)	8.7 (0.2)	17.8 (0.1)	16.1 (0.2)	18.2 (0.1)	16.6 (0.2)	18.2 (0.1)	16.6 (0.2)
	6.5	9.2 (0.1)	9.7 (0.1)	19.8 (0.1)	17.1 (0.1)	22.7 (0.1)	20.3 (0.1)	22.7 (0.1)	20.4 (0.1)
	10	8.9 (0.2)	9.5 (0.5)	21.1 (0.2)	15.5 (0.6)	25.0 (0.2)	19.3 (0.8)	25.0 (0.2)	19.4 (0.8)
0.4 × 10^{-4}	3.5	8.3 (0.1)	8.9 (0.2)	13.2 (0.1)	12.3 (0.2)	13.8 (0.1)	12.6 (0.3)	13.9 (0.1)	12.7 (0.3)
	6.5	8.8 (0.2)	9.3 (0.2)	15.8 (0.1)	14.0 (0.2)	19.7 (0.1)	16.2 (0.2)	19.8 (0.0)	16.2 (0.2)
	10	8.1 (0.1)	8.8 (0.1)	11.8 (0.1)	11.4 (0.1)	15.0 (0.1)	14.5 (0.1)	15.0 (0.1)	14.6 (0.1)
0.8 × 10^{-5}	3.5	8.8 (0.1)	9.4 (0.2)	-	-	11.1 (0.2)	10.2 (0.2)	11.1 (0.1)	10.3 (0.2)
	6.5	8.0 (0.1)	8.7 (0.1)	-	-	8.6 (0.1)	9.0 (0.1)	8.6 (0.1)	9.0 (0.1)
	10	8.7 (0.1)	9.6 (0.7)	-	-	11.3 (0.1)	12.4 (0.7)	11.3 (0.1)	12.4 (0.7)
1.1 × 10^{-4}	3.5	8.9 (0.2)	9.5 (0.2)	-	-	9.0 (0.2)	9.5 (0.1)	9.0 (0.2)	9.5 (0.1)
	6.5	8.2 (0.1)	8.9 (0.1)	-	-	8.4 (0.1)	9.1 (0.1)	8.4 (0.1)	9.1 (0.1)
	10	7.9 (0.1)	8.8 (0.1)	-	-	8.3 (0.1)	8.9 (0.1)	8.4 (0.1)	8.9 (0.1)
1.2 × 10^{-4}	3.5	8.8 (0.1)	9.4 (0.2)	-	-	8.8 (0.1)	9.4 (0.1)	8.8 (0.1)	9.4 (0.1)
	6.5	7.6 (0.1)	8.1 (0.1)	-	-	7.8 (0.2)	8.3 (0.1)	7.8 (0.2)	8.3 (0.1)
	10	8.8 (0.1)	9.9 (0.3)	-	-	9.2 (0.2)	10.1 (0.3)	9.2 (0.1)	10.1 (0.3)

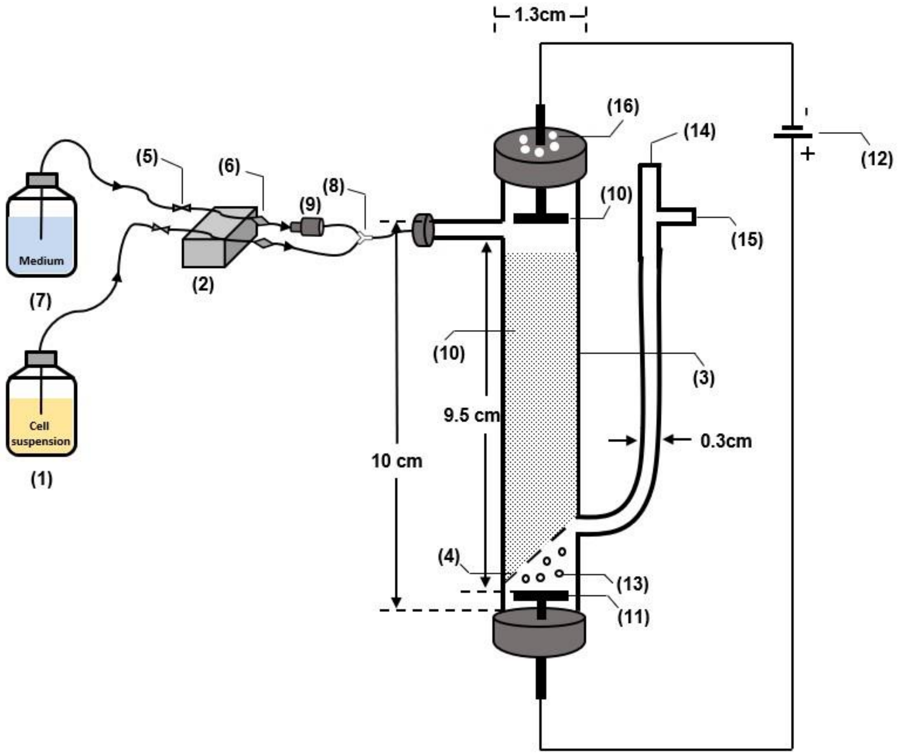

Figure S1. Schematic view of the percolation column setup used to assess the effect of DC electric fields on NAH biodegradation in porous media. It consists of a bottle (1) containing a bacterial suspension which was flushed by a peristaltic pump (2) from the top to the bottom of the column (3). The column was confined at the bottom by a glass frit (4). A water-stopped clip (5) was connected to the tubes with a connector (6) and a three-way pipe (8) to control the solution transport. The minimal medium in another bottle (7) was carried into a reservoir (9) filled with crystalline naphthalene for the generation of the solution of water dissolved NAH and then flowed through the column after bacterial attachment. The column was packed with a bed of small glass beads and contained two disk-shaped Ti/Li electrodes (10 and 11) at its top and bottom end. The electrodes were connected to a DC power pack (12) allowing for a constant DC electric field. To avoid an accumulation of gas released from the bottom electrode in the frit chamber, the oxygen bubbles (13) were allowed to discharge via a bypass glass tube (14), which simultaneously acted as a sampling port (15) for column effluents. Hydrogen bubbles (16) formed at the cathode were allowed to escape by an intended leakage at the top of the column.

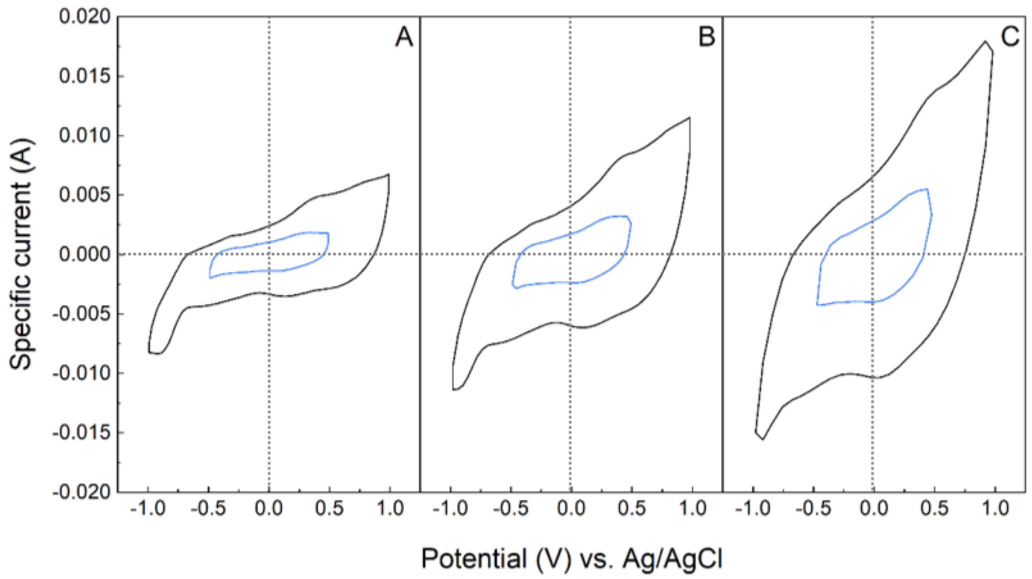

Figure S2. Cyclic voltammograms of NAH during electrolysis under different conditions. CV was performed in 100 mM PB at 1 mV s^{-1} (A); 2 mV/s (B); and 4 mV/s (C).

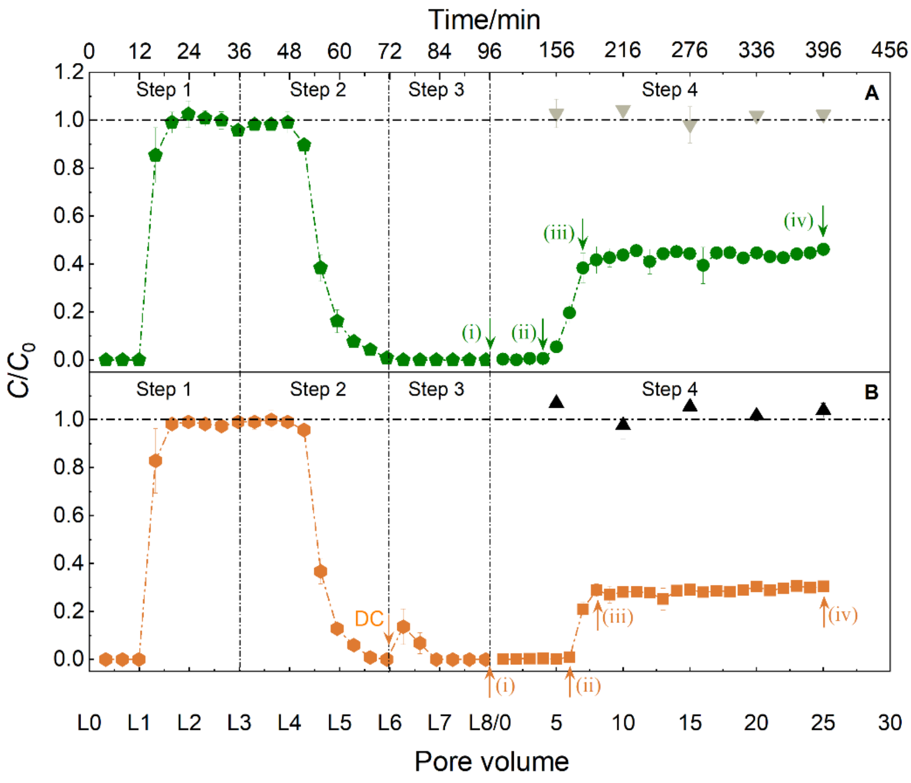

Figure S3. **Representative relative outflow concentrations of *P. fluorescens* LP6a cells (0 – 96 min; L0 – L8) and NAH (96 – 396 min; 0 – 25) in the absence (green, A) and presence of DC (*E* = 0.5 V·cm^{-1}; orange, B).** The inflow C_0 of the cell density and NAH were $OD_{600\,nm}$ ≈ 0.03 and 5.1 × 10^{-5} mol·L^{-1} flowing at $\overline{U}$ = 0.4 ×10^{-4} m·s^{-1}. The experiment was run in four steps. Step 1 (L0 – L3): Loading of columns for 3 PV. Step 2 (L3 – L6): flushing of columns with 3 PV of PB. Step 3 (L6 – L8): PB flushing during 2 PV either in the absence (control) or presence of DC (cf. arrow). Step 4 (0 – 25): inflow of NAH in PB containing nutrients. The points (i, ii, iii, iv) reflect times after loading (i), at point of increasing NAH outflow concentrations (ii), at begin of stable NAH outflow concentration (iii), and at the end of the experiment (iv), respectively.

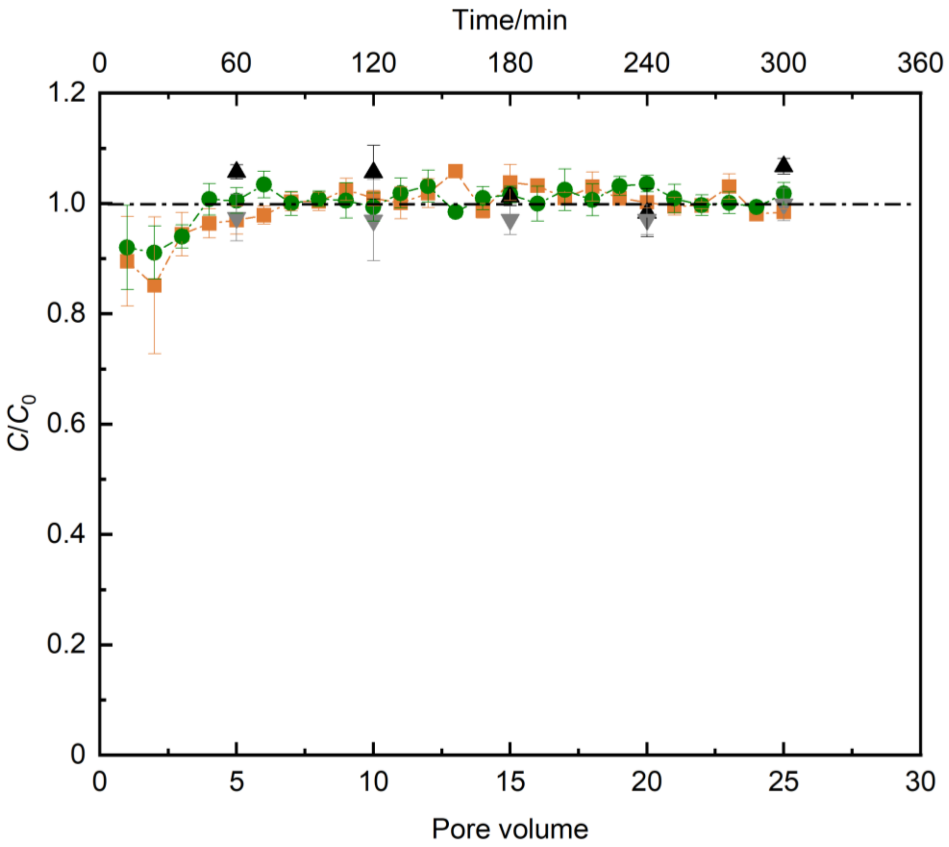

Figure S4. **Relative NAH outflow concentrations in columns with heat-inactivated *P. fluorescens* Lp6a cells in the absence (green) and presence of DC (*E* = 0.5 V·cm^{-1}; orange).** The inflow C_0 of NAH was 7.8×10^{-5} mol·L^{-1} flowing at $\overline{U} = 0.4 \times 10^{-4}$ m·s^{-1}.

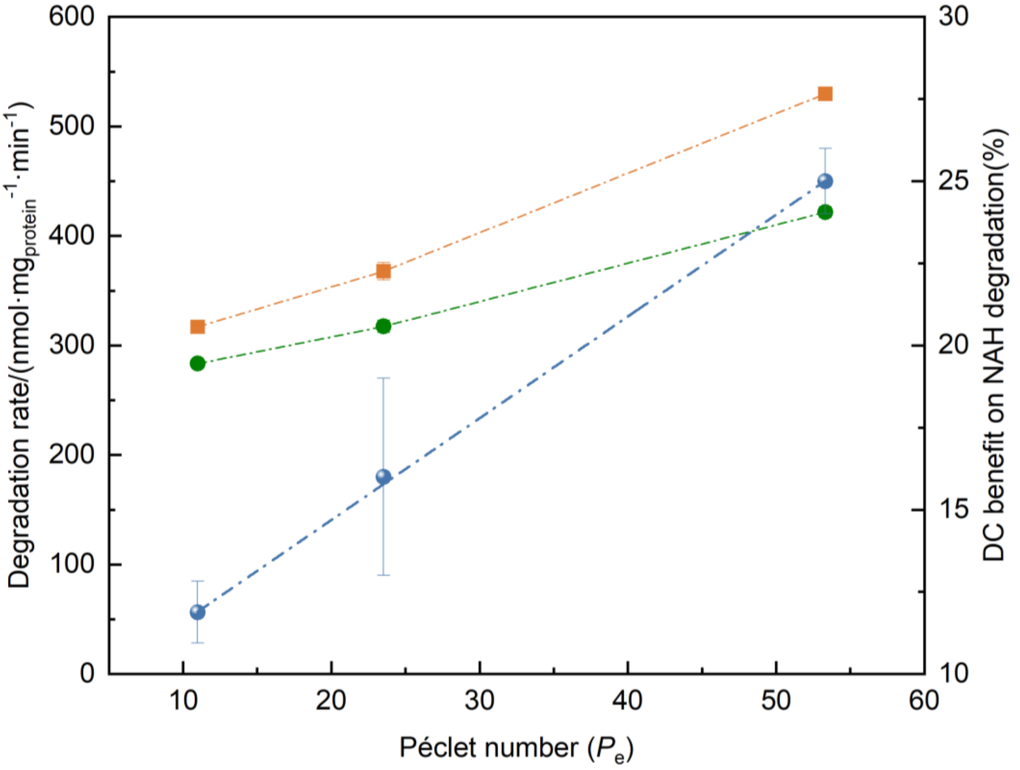

Figure S5. Effect of glass bead diameter-dependent Péclet numbers on NAH degradation rates (left y-axis) and DC-induced degradation benefits (right y-axis) in absence (green) and presence of DC at *E* = 0.5 V·cm^{-1} (orange). The inflow C_0 of NAH was 7.8 × 10^{-5} mol·L^{-1} flowing at $\overline{U}$ = 0.4 × 10^{-4} m·s^{-1}. The radii of the glass beads were 8.75 × 10^{-5} (P_e = 11), 1.8 × 10^{-4} m (P_e = 24), and 4.25 × 10^{-4} m (P_e = 53).

.

REFERENCES

(1) Johnson, W. P.; Blue, K. A.; Logan, B. E.; Arnold, R. G., Modeling Bacterial Detachment during Transport through Porous-Media as a Residence-Time-Dependent Process. *Water Resour. Res*. **1995**, 31(11), 2649-2658.

(2) Rabiei,P.; Mohabatkar, H.; Behbahani, M. Studying the Effects of Several Heat-inactivated Bacteria on Colon and Breast Cancer Cells. *Mol*. *Biol*. *Res. Commun*. **2019**, 8(2), 91-98.

(3) Sommerfeld, A. Ein Beitrag zur Hydrodynamischen Erkläerung der Turbulenten Flüssigkeitsbewegüngen (A Contribution to Hydrodynamic Explanation of Turbulent Fluid Motions). *International Congress of Mathematicians(ICM).* **1908**, 3, 116-124.

(4) Reynolds, O. An Experimental Investigation of the Circumstances which Determine whether the Motion of Water shall be Direct or Sinuous, and of the Law of Resistance in Parallel Channels. *Philos. Trans. Royal Soc. A.* **1883**, 174, 935-982.

(5) Gommes, C.; Tharakan, J. The Péclet Number of a Casino: Diffusion and Convection in a Gambling Context. *Am. J. Phys*. **2020**, 88 (6), 439-447.

(6) Simoni, S. F.; Schäfer, A.; Harms, H.; Zehnder, A. J. B. Factors Affecting Mass Transfer Limited Biodegradation in Saturated Porous Media. *J. Contam. Hydrol.* **2001**, 50, 99-120.

(7) Rice, C.L.; Whitehead, R. Electrokinetic Flow in a Narrow Cylindrical Capillary. *J. Phys. Chem.* **1965**, 69, 4017-4024.

(8) Shi, L.; Susann, M.; Harms, H.; Wick, L. Y. Factors Influencing the Electrokinetic Dispersion of PAH-Degrading Bacteria in a Laboratory Model Aquifer. *Appl. Microbiol. Biotechnol*. **2008**, 80 (3), 507-515.

(9) Ghosal, S. Fluid Mechanics of Electroosmotic Flow and Its Effect on Band Broadening in Capillary Electrophoresis. *Electrophoresis*. **2004**, 25 (2), 214−228.

(10) Sharma, P. K.; Rao, K. H. Adhesion of *Paenibacillus polymyxa* on Chalcopyrite and Pyrite: Surface Thermodynamics and Extended DLVO Theory. *Colloids Surf. B*. **2003**, 29(1), 21-38.
